# Increased SERCA2a sub-cellular heterogeneity in right-ventricular heart failure inhibits excitation-contraction coupling and modulates arrhythmogenic dynamics

**DOI:** 10.1098/rstb.2021.0317

**Published:** 2022-11-21

**Authors:** M. Holmes, M. E. Hurley, T. M. D. Sheard, A. P. Benson, I. Jayasinghe, M. A. Colman

**Affiliations:** ^1^ Faculty of Biological Sciences, University of Leeds, Leeds LS2 9JT, UK; ^2^ School of Biomedical Sciences, University of Leeds, Leeds LS2 9JT, UK; ^3^ Institute of Membrane and Systems Biology, University of Leeds, Leeds LS2 9JT, UK; ^4^ School of Biosciences, The University of Sheffield, Sheffield S10 2TN, UK

**Keywords:** calcium handling, excitation-contraction coupling, arrhythmia, heterogeneity, serca, heart failure

## Abstract

The intracellular calcium handling system of cardiomyocytes is responsible for controlling excitation-contraction coupling (ECC) and has been linked to pro-arrhythmogenic cellular phenomena in conditions such as heart failure (HF). SERCA2a, responsible for intracellular uptake, is a primary regulator of calcium homeostasis, and remodelling of its function has been proposed as a causal factor underlying cellular and tissue dysfunction in disease. Whereas adaptations to the global (i.e. whole-cell) expression of SERCA2a have been previously investigated in the context of multiple diseases, the role of its spatial profile in the sub-cellular volume has yet to be elucidated. We present an approach to characterize the sub-cellular heterogeneity of SERCA2a and apply this approach to quantify adaptations to the length-scale of heterogeneity (the distance over which expression is correlated) associated with right-ventricular (RV)-HF. These characterizations informed simulations to predict the functional implications of this heterogeneity, and its remodelling in disease, on ECC, the dynamics of calcium-transient alternans and the emergence of spontaneous triggered activity. Image analysis reveals that RV-HF is associated with an increase in length-scale and its inter-cellular variability; simulations predict that this increase in length-scale can reduce ECC and critically modulate the vulnerability to both alternans and triggered activity.

This article is part of the theme issue ‘The cardiomyocyte: new revelations on the interplay between architecture and function in growth, health, and disease’.

## 1. Introduction

The intracellular calcium (Ca^2+^) handling system of cardiomyocytes links cellular electrical and mechanical function [1], referred to as excitation-contraction coupling (ECC). Ca^2+^ homeostasis is regulated by the balance of intracellular Ca^2+^ fluxes through specialized ion channels and transporters. Maintaining normal Ca^2+^ homeostasis is critical to the contractile performance of the heart, ensuring a stable cardiac output able to meet the body's dynamic physiological demands [2,3], as well as preventing Ca^2+^-overload which can lead to cell death and other pathophysiological phenomena.

Ca^2+^ homeostasis is initially conferred in the dyadic cleft by the process of Ca^2+^-induced-Ca^2+^-release (CICR), wherein a Ca^2+^ influx through the sarcolemmal L-type-Ca^2+^-channels (LTCCs) during electrical excitation triggers the type-2 ryanodine receptors (RyRs) to release Ca^2+^ from the intracellular Ca^2+^ store, the sarcoplasmic reticulum (SR). Diastolic Ca^2+^ concentrations are restored by the Na^+^-Ca^2+^ exchanger (NCX), responsible for extracellular efflux, and the SR-Ca^2+^-pump (SERCA2a), responsible for refilling the SR in preparation for the next systolic cycle. Abnormalities in Ca^2+^ homeostasis have been linked to deficiencies in SERCA2a function [4], and aberrations in ECC are associated with the development of pro-arrhythmogenic cellular dynamics, including Ca^2+^-transient alternans and arrhythmia triggers [3,5]. Dissecting the multi-scale mechanisms underlying these pathophysiological dynamics is crucial to understanding the development of cardiac arrhythmias.

The general mechanisms of ECC are well documented and understood [1,2]. However, recent studies highlight gaps in our understanding of the relationships between sub-cellular structure (i.e. the spatial arrangement and co-localization of the multiple Ca^2+^ transporters) and the function of the intracellular Ca^2+^ handling system [6,7]. Heterogeneity in the expression of RyR, SERCA2a and NCX throughout the sub-cellular volume has been indicated in multiple experimental imaging studies [8–10]; the role and importance of this heterogeneity in maintaining normal function has yet to be elucidated. Moreover, many cardiac conditions such as heart failure (HF) are associated with remodelling of the expression of these Ca^2+^ transporters as well as sub-cellular structure [10–13], most notably the transverse and axial tubular system (T-system), responsible for delivering the electrical action potential (AP) to the cell interior to induce cell wide and uniform CICR. It is unclear whether concomitant remodelling of the sub-cellular heterogeneity in the Ca^2+^ transporters occurs and, if so, whether such remodelling is pro-arrhythmogenic or protective.

Computational modelling is a powerful tool to dissect the mechanisms underlying cardiac function in health and disease, through enabling the isolation of individual components and specific changes within a system [14,15]. Sophisticated models of spatio-temporal Ca^2+^ handling have been developed over the last decade, for example, accounting for the spatial distribution of cardiac dyads, gating stochasticity in RyRs and LTCCs [7,9,16–18], heterogeneous expression in dyadic properties [19] and realistic sub-cellular structure [7,18,20]. These recent advances enable image-based modelling to be performed, bridging the gap between experiment and simulation and revealing the underlying details of the governing structure–function relationships.

Previous quantification of heterogeneous expression in SERCA2a has been limited, both in normal and in remodelled hearts. In this study, a novel technique [21] to quantify heterogeneous SERCA2a expression in the sub-cellular volume was applied to analyse previously collected imaging data from healthy and failing cardiomyocytes. Image-based computational modelling was then applied to assess the implications of observed heterogeneity, and its remodelling in disease, on cellular function and inter-cellular variability.

## 2. Methods

### (a) Image analysis

Previously published [22] and unpublished confocal resolution microscopy images of SERCA2a expression in rat ventricular myocytes were analysed. The animal models followed a well-established protocol for monocrotaline (MCT)-induced pulmonary hypertension [23–25]. Adult male Wistar rats weighing 180–215 g were administered an intraperitoneal injection of either saline solution (140 mM NaCl) or MCT (Sigma Aldritch, 60 mg kg^−1^). The development of right-ventricular (RV) hypertrophy over the course of four weeks leads to RV-HF [26]. Animals underwent schedule 1 (euthanasia) by concussion followed by cervical dislocation when signs of HF were evident. Control animals were taken as day-matched for the MCT-treated animals.

Following cell isolation, myocytes were fixated *in situ*, permeabilized with 0.1% Triton X-100 and blocked with 10% normal goat serum in phosphate buffer saline at room temperature, labelled for SERCA2a and then imaged using a LSM880 Inverted microscope (Carl Zeiss, Jena; full description in the electronic supplementary material). Quantifying the spatial profile of SERCA2 from these microscopy data required the construction of a semi-automatic pipeline which processed the data into a suitable format for analysis and a method of fitting the processed data to some spatial covariance function [21]. The aim of this analysis is to extract the length-scale, *λ*, which describes the distance over which expression is correlated. A long length-scale means that expression is correlated over large distances, corresponding to smooth spatial variation between large regions of high and low expression; a short length-scale means that expression is not correlated over large distances, corresponding to spatially rapid gradients between small regions of expression. We are interested in how the expression of SERCA2a varies between different regions of the cell, rather than super-resolution features such as co-localization distances, and hence consider distances at 1 micron or larger, by averaging SERCA2a expression over 1-micron voxels. A length-scale of 1 µm therefore corresponds to no-spatial correlation, where the expression in each 1-micron voxel is independent of its neighbours. This down-sampling is also necessary owing to the requirement for continuous spatial data: SERCA2a follows the structure of the SR, and so it is not spatially continuous at the high resolution of the original images. More sophisticated analysis methods would need to be developed in order to extract the length-scale along the SR structure. Down-sampling removes this structure and leaves only the average, continuous expression in 1-micron voxels, enabling the length-scale to be accurately extracted above these distances.

**Figure 1.**
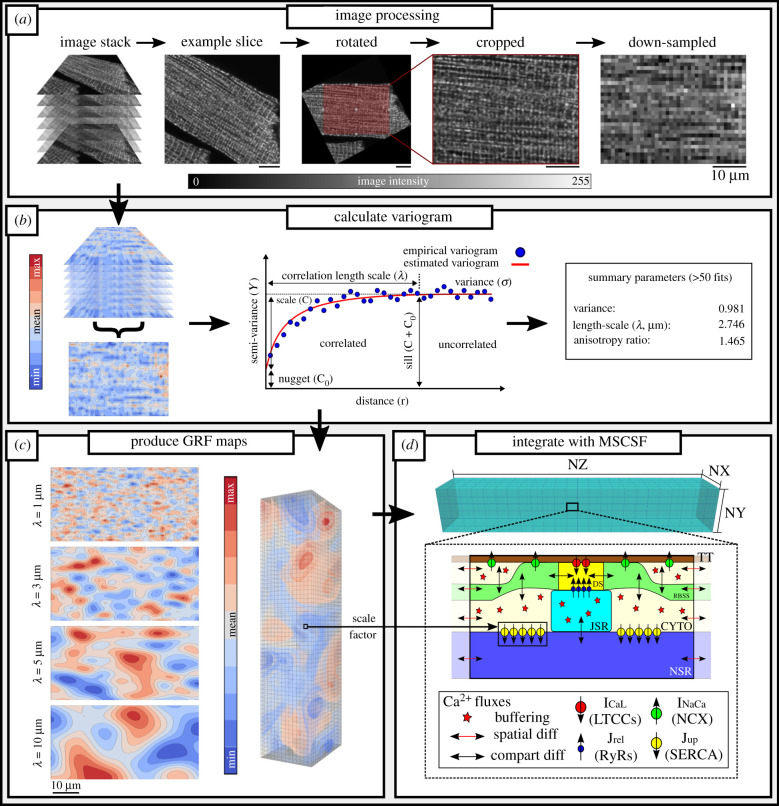
Image processing and experimental-simulation framework. (*a*) Original microscopy images are semi-automatically processed into a form suitable for a variogram fitting protocol, rotating the longitudinal axis to match the *x*-axis, cropping and down-sampling. Scale bar illustrates 10 µm for all panels. (*b*) The spatial variation across pairs of points in the integrated two-dimensional dataset is plotted as a function of distance (a variogram) to measure the length-scale of correlation in the spatial data. Outputs are a statistical summary of greater than 50 successful fits. (*c*) Examples of two-dimensional Gaussian random field (GRF) maps produced at different length-scales (left) and an illustration of a full three-dimensional GRF map (right). (*d*) Fundamental structure of the three-dimensional spatio-temporal Ca^2+^ handling model in the multi-scale cardiac simulation framework (MSCSF) [27], illustrating the compartments for each calcium release unit (CRU) of the dyadic cleft (DS), reduced-buffering subspace (RBSS), bulk cytoplasmic space (CYTO) and junctional and network SR (JSR and NSR, respectively), as well as the primary Ca^2+^ fluxes. The GRF map determines the local scale factor for the maximal flux-rate, $J_{\mathrm{up}}^{\max}$, representing SERCA2a expression in each CRU.

Each cell was orientated such that the *z*-lines were orthogonal to the transversal axis. The most suitable section of the image was selected such that the analysis excluded any image background, fragments or nuclei, before being down-sampled to a resolution of 1 µm (figure 1*a*). The same processing parameters were applied to each cell within the stack before the images are integrated over the *z*-axis (cell depth), condensing the data into a two-dimensional image for the experimental variogram fitting procedure (figure 1*b*).

An empirical variogram was estimated for each of these processed datasets using an algorithm which calculated the following equation:

2.1 $$\begin{array}{c} \gamma (r_{k})=\frac{1}{2N(r_{k})}\overset{N(r_{k})}{\underset{i=1}{\sum }}{\left(z(x_{i})-\;z(x_{i}^{\prime })\right)}^{2}\;, \end{array}$$

where *N(r*_*k*_) is the total number of bins, *z*(*x*) is the value of the field at point *x* and *γ*(*r*_*k*_) is the empirical semi-variance computed using the distance bins, *r*_*k*_, a measurement of the spatial dependency between all sets of two points $\left(x,x^{\prime }\right)$ at some distance *r*, and the bins are given by $r_{k}\leq x_{i}-x_{i}^{\prime }\;<r_{k+1}$. The estimated variogram was then fitted using some covariance function to estimate correlation length-scales (figure 1*b*) [28]. This is given by the general formula

2.2 $$\begin{array}{c} \gamma (r)=\;\sigma^{2}\cdot (1-\mathrm{cor}(r))+C_{0}, \end{array}$$

where γ(r) is the general semi-variance for a distance *r*, $\sigma^{2}$ is variance and *C*_0_ is the nugget, the height of discontinuity at the origin, representing a non-zero variance at *r* = 1 (figure 1*b*) and $\sigma^{2}\cdot (1-\mathrm{cor}(r))$ is replaced by the specific covariance function. We used the squared exponential covariance function (also known as the Gaussian or SE kernel) as it is well suited to imaging studies owing to its stationarity and simplicity, this is given by:

2.3 $$\begin{array}{c} \mathrm{cov}(r)=k_{\mathrm{SE}}(x,x^{\prime })=\sigma^{2}e^{-{\left(x-x^{\prime }\right)}^{2}/2\lambda^{2}}\; \end{array},$$

where this expression replaces $\sigma^{2}\cdot (1-\mathrm{cor}(r))$ in equation (2.2), *k*_SE_ is the squared exponential (or Gaussian) kernel and λ is the correlation length-scale. The Gaussian kernel requires a smooth sample path to estimate this length-scale reliably, which is provided already through the process of down-sampling. This fitting was successfully performed a minimum of 50 times for three separate processed regions of each cell using a set of suitable binning parameters based on the final dimensions of the processed dataset. The results from each of these fittings in all three regions were used to produce a final quantification of a cell's spatial parameters.

### (b) Computational models

This study used a simplified version of the O'Hara-Rudy dynamic human ventricular model [27,29,30], integrated into our multi-scale cardiac simulation framework (MSCSF) compartmentalized Ca^2+^ dynamics model [27]. SERCA2a expression heterogeneity was imposed by applying a local scale factor to the maximal pump-rate for intracellular uptake, $J_{\mathrm{up}}^{\max}$, in each calcium release unit (CRU).

**Figure 2.**
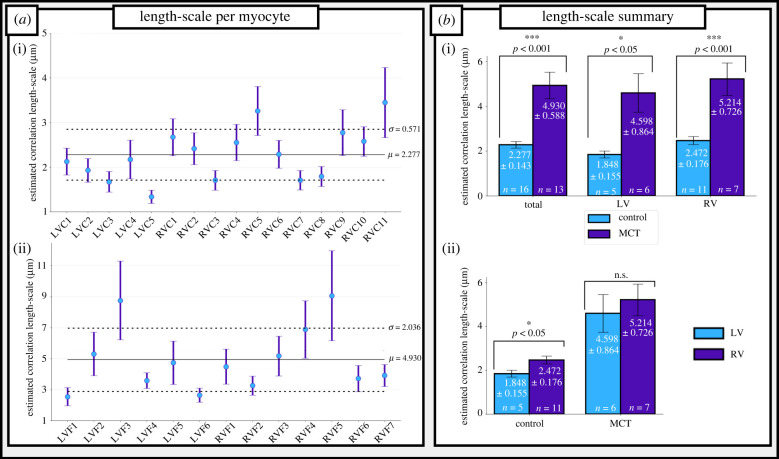
SERCA2a heterogeneity in control and RV-HF cardiomyocytes. (*a*) The comparison of cell-average correlation length-scales, *λ*, in control (saline-treated) (i) cells and monocrotaline (MCT)-treated cells (ii). Horizontal lines represent overall mean (full line) and s.d. (dotted line) for control and MCT cells, respectively. Vertical error lines are 95% confidence intervals. (*b*) Comparison of mean *λ* in control and MCT-treated rat ventricular myocytes (i) and in left ventricular (LV) and right ventricular (RV) cells (ii). (Online version in colour.)

Given the distribution of SERCA2a expression observed (see Results, figure 2), we assume that the spatial profile of SERCA2a within a cardiomyocyte can be modelled as a spatial random field—a function *f(x)* over a multi-dimensional space in which each point *x* ∈ ℝ^n^ takes some random value from a domain of real numbers [31,32]. A distribution function, such as the Gaussian probability density function, may be applied to a spatial random field (Gaussian random field, GRF) to impose constraints on variance, *σ*^2^, and correlation length-scale, *λ*, to reflect physiological boundaries on these parameters [33]. Thus, the length-scales extracted from the imaging data can directly inform the parameters of these randomly generated three-dimensional spatial fields (figure 1*c*), which can be produced at a discretization resolution corresponding to that of the three-dimensional computational model: each voxel (*N*_total_ = 19500, *N*_X_ = 15, *N*_Y_ = 20, *N*_Z_ = 65) of the spatial map represents one CRU, and the local *J*_up_^max^ scale factor is given by the associated value in the expression map (figure 1*d*).

It was observed (electronic supplementary material, figure S1) that whereas some small inter-cell variation in relative whole-cell SERCA2a expression was present, the significant difference was in correlation length-scales. In order to isolate the impact of length-scale only, isotropic maps (i.e. the same length-scale in the longitudinal and transverse directions) were generated with the same whole-cell mean SERCA2a expression and whole-cell input variance, *σ*^2^ = 1, at four correlation length-scales (*λ* = 1, 3, 5, 10 µm; e.g. figure 1*c*). Any number of unique GRFs may be produced using the same parameter set, enabling an assessment of the impact of structural arrangement on Ca^2+^ handling dynamics; except where otherwise stated, three independent maps were generated and implemented at each length-scale.

It is important to note that the length-scale and the total degree of heterogeneity/variation are independent of each other; the length-scale determines the spatial correlation of the values within the range defined by the total variation of the distribution. The degree of heterogeneity can be controlled by the standard deviation (*σ*) of the distribution (with a mean of 1.0, as we are interested in scale factors rather than absolute expression) or, equivalently, by the range defined by ±3*σ*. In the present study, a *σ* of 0.3 was implemented for all simulations at all length-scales, corresponding to the ±3*σ* range of 0.1–1.9. Note that this rescaling was applied after the normalized (*σ*^2^ = 1) GRF was produced.

### (c) Experimental and simulation protocols

In this study, we quantified the length-scales describing the sub-cellular distribution of SERCA2a in rat healthy and RV-failure myocytes. Simulations were then performed to assess the impact of different heterogeneity parameter profiles on the intracellular Ca^2+^ transient (CaT) under normal pacing and pro-arrhythmogenic conditions, corresponding to CaT alternans and spontaneous Ca^2+^-release events (SCRE).

#### (i) Normal pacing and rate dependence

Expression maps were loaded into the MSCSF [27] and paced for 60 beats at steady state at rates of 60, 75, 120, 133 and 150 beats per minute (bpm). At each of the selected correlation length-scales (*λ* = 1, 3, 5, 10 µm), three maps were used for a total of 12 heterogeneous maps and the homogeneous control.

#### (ii) Rapid pacing, sarcoplasmic reticulum loading and alternans

Ca^2+^ transient alternans were studied by applying rapid pacing in combination with multiple parameter combinations which are known to promote alternans [7,34]: namely, a reduction to the activity of the LTCCs (through either a reduction to the channel expression, corresponding to number of channels per dyad, *N*_LTCC_, or a reduction to the channel open transition rate, LTCC_PO­_) and SERCA2a (reduction to the global parameter for $J_{\mathrm{up}}^{\max}$), individually and combined. Global changes were applied consistently to both homogeneous and heterogeneous sub-cellular SERCA2a expression maps.

In order to induce SR-Ca^2+^ loading which promotes the emergence of SCRE (and thusly pro-arrhythmogenic triggers), rapid pacing (basic cycle length = 400 ms) was applied in combination with a functional model of isoprenaline (ISO) which comprises enhanced LTCC activity (×2) and SERCA2a activity (×1.75) as well as enhanced K^+^-currents to maintain AP duration [27]. Owing to the importance of SERCA2a for SR-Ca^2+^ loading and the uncertainty in the degree of enhanced activity owing to ISO, we also introduced a condition in which $J_{\mathrm{up}}^{\max}$ was further upregulated by a factor of 1.5 in combination with ISO. To enable statistical analysis, 20 simulations were performed for each heterogeneous map for each condition, and 50 simulations were performed with the homogeneous, control map.

## 3. Results

### (a) Length-scale of SERCA2a heterogeneity is increased in right-ventricular heart failure

In total, 29 datasets were analysed, including stacks and single images from both left ventricular (LV) and RV rat cardiomyocytes taken from animals which underwent the control (saline injection; *N* myocytes = 5 LV and 11 RV) and MCT (*N* = 6 LV and 7 RV) treatment.

Correlation length-scales, *λ*, were observed (figure 2*a*) to range between 1 and 4 µm in control cells (mean = 2.277 ± 0.143 µm) and between 2 and 11 µm in MCT cells (mean = 4.930 ± 0.588 µm). A significant difference in *λ* of these cells was found (figure 2*b*; *p* < 0.001), thus the observed remodelling in HF [10–13] has the effect of increasing the spatial correlation of SERCA2a in the sub-cellular volume (larger length-scales) as well as increasing inter-cellular variability (larger range of length-scales). This significance is also present when isolating LV and RV cells (figure 2*b*): RV cells were observed to have a higher correlation length-scale in both control and HF remodelling (control: mean = 2.472 ± 0.176 µm; MCT: mean = 5.214 ± 0.864 µm; *p* < 0.001) than LV cells (control: mean = 1.848 ± 0.155 µm; MCT: mean = 4.598 ± 0.864 µm; *p* < 0.05). Throughout this paper, an increase in length-scale is considered as an increase in heterogeneity, as it corresponds to larger patches of high/low channel expression, although we recognize that ‘increased heterogeneity’ can be ambiguous in these cases. Thus, from herein, ‘increased heterogeneity’ is synonymous with an increase in *λ*.

### (b) Increased length-scale in SERCA2a heterogeneity reduces the magnitude and increases the spatial variation of the Ca^2+^ transient

**Figure 3.**
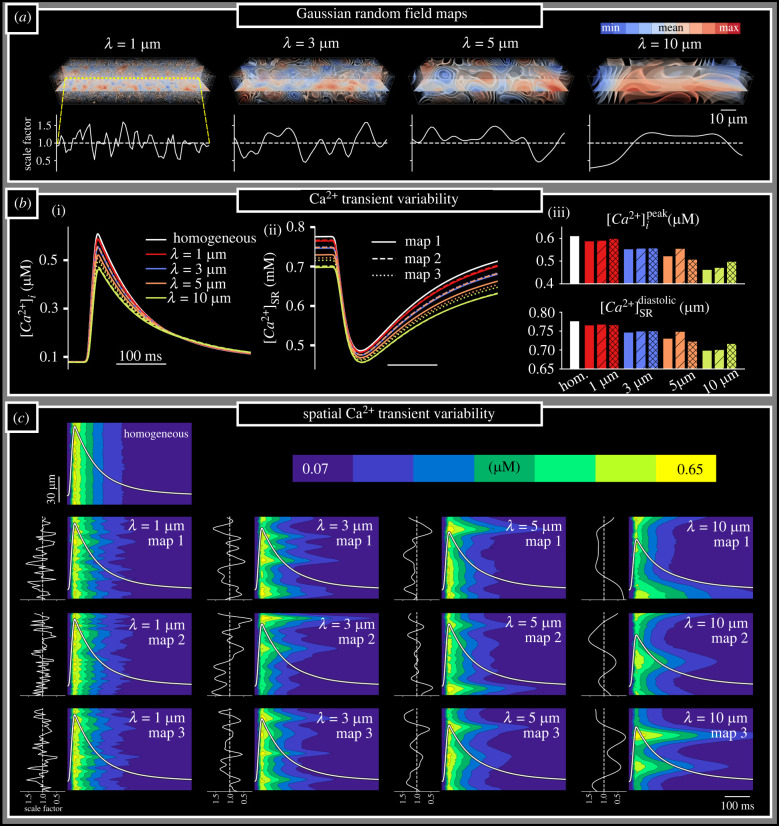
Ca^2+^ transient variability as a consequence of SERCA2a heterogeneity. (*a*) Illustration of the GRF maps, describing the local $J_{\mathrm{up}}^{\max}$ scale factor, produced at different length-scales (upper). The value of the scale factor along a longitudinal linescan through the centre of the cell is shown for clarity (lower). Maps shown correspond to ‘map 1’ of the three independent GRF maps used at each length-scale. (*b*) Whole-cell Ca^2+^ transients (i) and SR-Ca^2+^ (ii) during normal pacing, for homogeneous control (white) and heterogeneous SERCA2a maps at each length-scale (1 µm—red; 3 µm—blue; 5 µm—orange; 10 µm—yellow); three maps at each length-scale were used (solid, dashed and dotted lines). Summary of the CaT magnitude (iii) and diastolic SR-Ca^2+^ (iv) at each length-scale and for each map. (*c*) Space–time images of the Ca^2+^ transient in the longitudinal axis (through the centre of the cell) in the homogeneous and all heterogeneous map conditions, corresponding to the same normal pacing excitations as shown in (*b*). Normalized whole-cell-average CaTs are overlaid in white for context. Plots to the left of each space–time image show the SERCA2a scale factor along the same longitudinal linescan (as illustrated in (*a*)).

The length-scale parameter input had a clear impact on the spatial properties of the three-dimensional GRFs used to perform simulations (figure 3*a*), congruent with the expectations of correlation length-scales. In control pacing, whole-cell CaTs (obtained by averaging the local concentrations in each CRU across the cell) generally decreased in magnitude as length-scale increased (figure 3*b*) despite the maintained global expression of SERCA2a. The reduction in the CaT was ultimately attributable to the diastolic SR-Ca^2+^ load, which was significantly reduced compared to the homogeneous condition as length-scale increases (figure 3*b*). An increase in length-scale was also associated with an increase in inter-cellular variability of both the CaT magnitude and diastolic SR-Ca^2+^ load.

The reduction in CaT magnitude associated with longer length-scales is accompanied by an increase in the spatial variation of the CaT throughout the cell, as a direct consequence of local SERCA2a function: regions of high/low SERCA2a spatially correlated with more rapid/slower decay of the CaT (figure 3*c*). This spatial variation, and its inter-cellular variability, was increased with length-scale.

**Figure 4.**
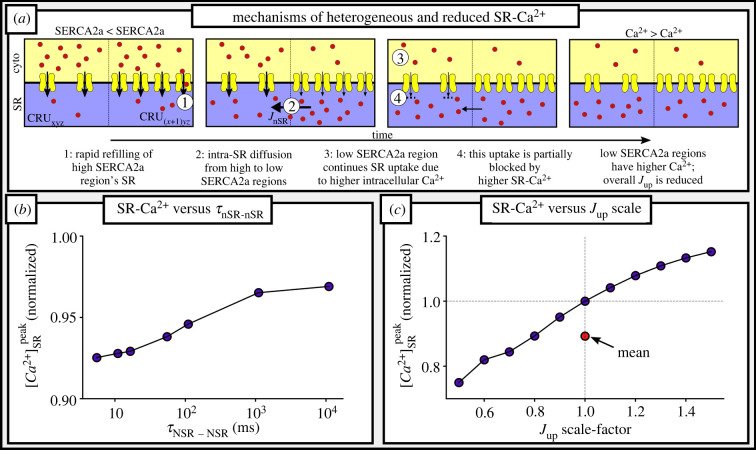
Mechanisms of heterogeneous and reduced SR-Ca^2+^. (*a*) Cartoon illustration of the mechanisms by which low-SERCA2a regions may exhibit the highest peak diastolic SR-Ca^2+^ during regular pacing. (*b*) The relationship between the time-constant of intra-SR diffusion (*τ*_nSR-nSR_, shown on a log-scale) in a single heterogeneous map and diastolic peak SR-Ca^2+^, normalized to the value in the homogeneous cell model under control conditions. (*c*) The relationship between global *J*_up_ scale factor (SERCA2a expression) in homogeneous cell models and diastolic peak SR-Ca^2+^, normalized to the value in the homogeneous cell model under control conditions. The red marker indicates the mean across the scale factors. (Online version in colour.)

The spatial profile of SR-Ca^2+^ did not exhibit such a simple relation to local SERCA2a expression and was dependent on the time within the cycle (figure 4*a*; electronic supplementary material, figure S2): during early refilling stages, the regions with high SERCA2a expression exhibited the most rapid refilling and thus larger local SR-Ca^2+^; however, by late-stage refilling towards the end of the cycle, regions of low-SERCA2a expression exhibited the highest SR-Ca^2+^ load owing to a combination of diffusion within the SR (from high to low-SERCA2a regions) and continued uptake in these low-SERCA2a regions owing to local cytosolic Ca^2+^ remaining high (figure 4*a*). The increased SR-Ca^2+^ load in these regions should reduce the activity of *J*_up_ relative to this cytosolic Ca^2+^, providing a potential mechanism by which SR diffusion can reduce the overall activity of *J*_up_ in heterogeneous map conditions.

The impact of intra-SR diffusion on cell-averaged SR-Ca^2+^ (figure 4*b*) was evaluated by varying the time-constant of diffusion within the SR in a single heterogeneous SERCA2a map (map 1 at a length-scale of 5 µm). Larger time-constants (i.e. slower diffusion) led to a smaller reduction in the SR-Ca^2+^ relative to the homogeneous model than smaller time-constants, supporting the feasibility of the above proposed mechanism. However, even at time-constants that effectively block SR diffusion within the time-scale of the cardiac cycle, the SR-Ca^2+^ load was still reduced compared to the homogeneous control, indicating that this mechanism alone does not fully explain the observations. Nonlinearity in the dependence of *J*_up_ on both cytosolic- and SR-Ca^2+^ (electronic supplementary material, figure S3) could also contribute. This was evaluated by assessing the diastolic SR-Ca^2+^ load in homogeneous cell models with the global expression of SERCA2a scaled (figure 4*c*): increasing SERCA2a expression led to a relatively smaller increase in diastolic SR-Ca^2+^ than the reduction that was observed when SERCA2a was reduced (figure 4*c*). Thus, averaging a normally distributed variation in SERCA2a would decrease the SR-Ca^2+^ load compared to the homogeneous, control condition; indeed, this is the case in the simulations where SR diffusion was effectively blocked. These two factors therefore combine to produce the overall observed reduction in SR-Ca^2+^ and consequently the CaT magnitude.

### (c) Rate dependence

SERCA2a heterogeneity had a negligible impact on the rate dependence of the AP (electronic supplementary material, figure S4). Properties of the Ca^2+^ handling system were more substantially affected by pacing rate, with the differences between homogeneous and heterogeneous conditions generally enhanced at rapid pacing rates compared to slower pacing rates (electronic supplementary material, figure S5). The nature of the rate dependence can also be affected: in homogeneous conditions, there was observed an increase in the CaT peak at more rapid pacing rates, whereas in the heterogeneous maps, pacing rates above 130 bpm demonstrated a reduction in the CaT peak.

### (d) Heterogeneous SERCA2a expression both promotes and inhibits alternans

**Figure 5.**
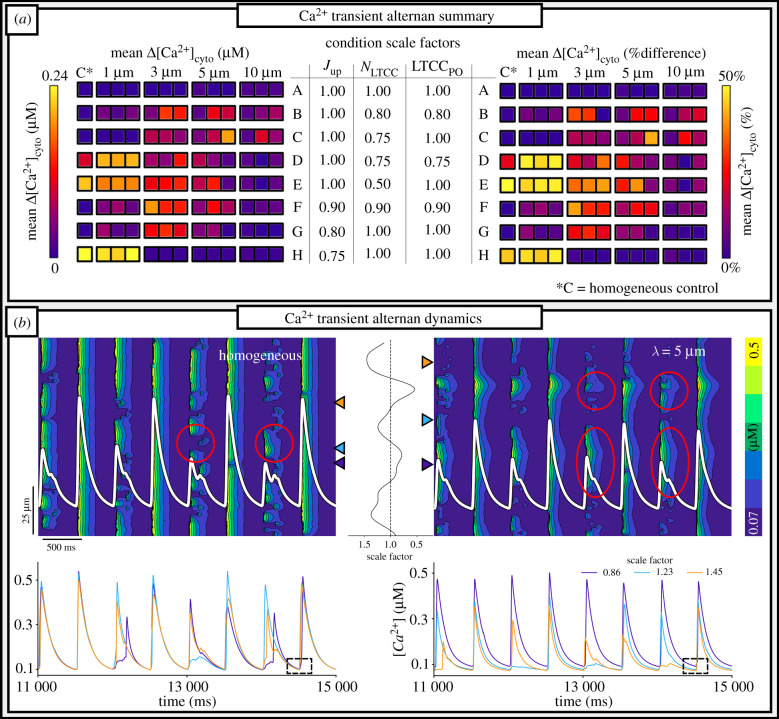
Summary of alternans behaviour in control to pro-arrhythmic conditions. (*a*) Colourmaps detailing the mean beat-to-beat difference in cytosolic Ca^2+^ (left) and percentage difference (right) in a range of conditions from control (*a*) to pro-arrhythmic (*b–h*) at 120 bpm. Conditions are described by scaling factors applied to each of $J_{\mathrm{up}}^{\max}$ (whole-cell SERCA2a flux), *N*_LTCC_ (L-type-Ca^2+^ channel density) and LTCC_Po_ (channel opening transition rate). Maps are organized left to right. (*b*) Space–time plots showing CaT alternans in two different conditions (upper). Whole-cell CaT is overlaid in white for context. Circled regions illustrate those which show either different behaviour on a beat-to-beat basis for the small beat (homogeneous, (i)) or broadly the same behaviour for the small beat (heterogeneous, (ii)). Lower panels show the local CaT at three selected CRUs for the same two conditions; coloured triangular markers indicate the location of each CRU selected for the plot. In the heterogeneous condition, each CRU has its own corresponding $J_{\mathrm{up}}^{\max}$ scale factor, indicated by the colour key. The dotted square highlights the same diastolic Ca^2+^ in the homogeneous condition and different diastolic Ca^2+^ in the heterogeneous condition.

Introducing heterogeneous underlying SERCA2a expression maps either inhibited alternans present in the homogeneous model, or induced alternans under conditions where they were not present in the homogeneous model. This shift (alternans to no alternans, or no alternans to alternans) occurs generally across the range of parameter combinations considered (figure 5*a*), although the fewest/smallest alternans were observed at a length-scale of 10 µm across all conditions. There was also a substantial degree of inter-map variation at each length-scale (especially 3–5 µm), i.e. the magnitude of alternans, and indeed whether or not they appeared, was dependent not only on the length-scale but also the specific features of the map, leading to increased inter-cellular variability. Despite the difference between some of the parameter combinations being very small (e.g. conditions G and H differ by only an additional 5% reduction in global SERCA2a), these disparities could lead to opposing behaviour. This indicates the high sensitivity of the emergence of alternans to model conditions and provides an explanation for the impact of SERCA2a heterogeneity: it can either push the cell into or out of the phase-space necessary for alternans, thus either inducing them where they were not present, or inhibiting them where they were present.

Whereas alternans in homogeneous cells demonstrated essentially random spatial properties (i.e. those CRUs which are active for the small beat vary on a beat-to-beat basis), the introduction of SERCA2a heterogeneity reduced the random nature of the alternans and introduced a broadly regular structure (figure 5*b*): those regions which were or were not active during the small beat were largely consistent across subsequent small beats. Analysis of the local CaT in individual CRUs reveals that this regularity is primarily determined by local diastolic Ca^2+^ (figure 5*b*) and local SR-Ca^2+^ (electronic supplementary material, figure S6): in regions of low SERCA2a, intracellular uptake is slow and thus decay of the CaT is slow; local diastolic Ca^2+^ therefore remains higher at the time of the next excitation, and, as with normal pacing, this is associated with higher local SR-Ca^2+^ loads; the RyRs are therefore more robust to reactivation. In the homogeneous model, however, there is no significant regular variation in diastolic Ca^2+^ or SR-Ca^2+^ throughout the cell and thus the alternans mechanism in this condition is not directly determined by local Ca^2+^.

### (e) Heterogeneous SERCA2a expression has a biphasic impact on spontaneous Ca^2+^-release events

Following the application of the rapid pacing SR-Ca^2+^ loading protocols described above, SCRE activity was detected by measuring characteristics of any wave exceeding a suitable threshold (greater than 0.135 µM) in cytosolic Ca^2+^ over the quiescent period. Delayed after depolarizations (DADs) and triggered action potentials (TA) were detected by measuring characteristics of any depolarization in transmembrane potential which exceeded suitable thresholds for each type of behaviour (greater than 1 mV deviation from the resting potential for a DAD; above −20 mV for TA).

**Figure 6.**
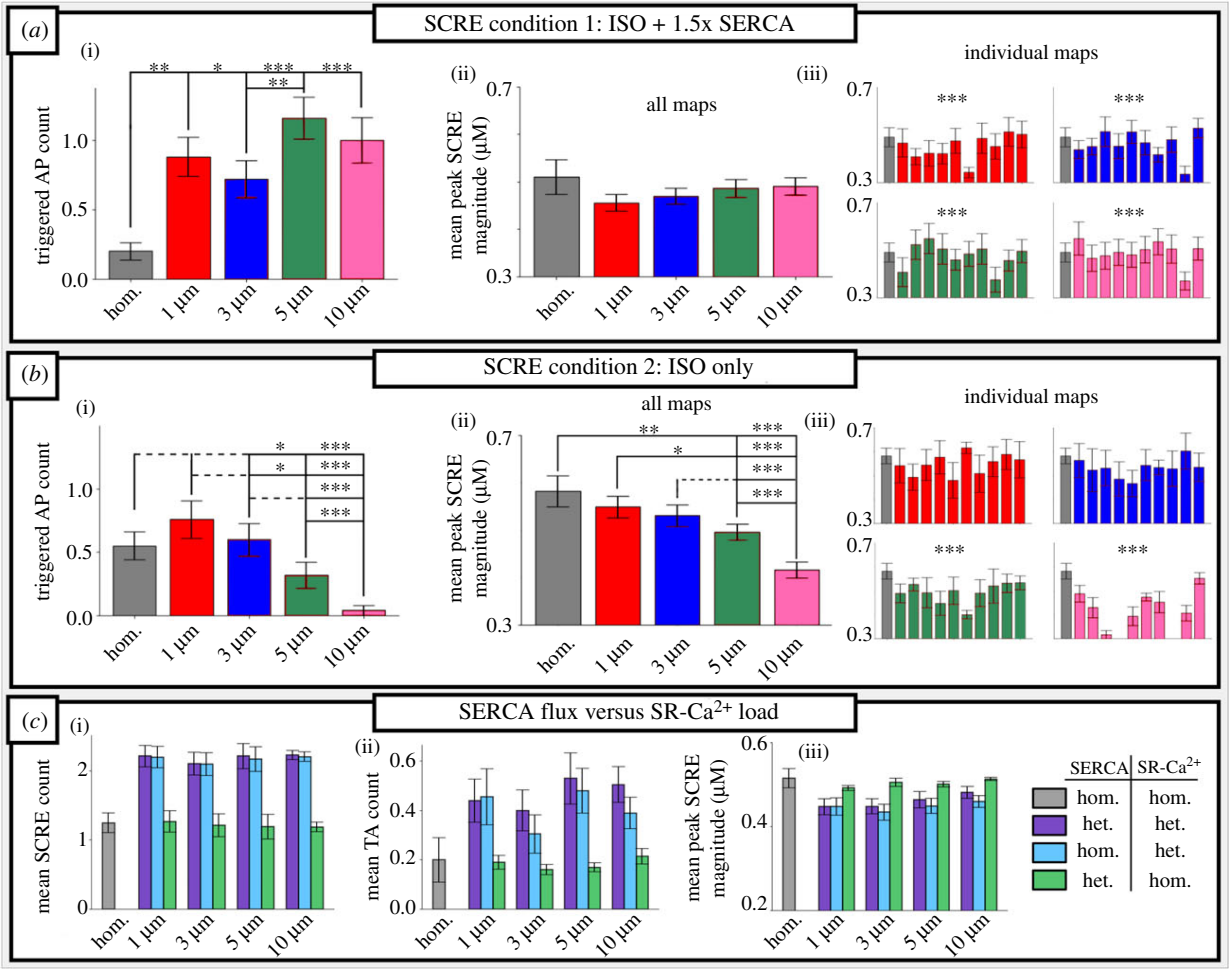
Impact of SERCA2a heterogeneity on spontaneous calcium release events. Statistical summary of SCRE behaviour for condition 1 (*a*) and condition 2 (*b*), showing triggered AP count (i) and mean peak SCRE CaT magnitude across all simulations (ii) and for each individual map (iii). (*c*) The comparison of mean SCRE count (i), TA count (ii) and SCRE magnitude (iii) for different combinations of heterogeneous/homogeneous SERCA2a maps and SR-Ca^2+^ load. (Online version in colour.)

In condition 1 (ISO + additional SERCA2a increase), the introduction of SERCA2a heterogeneity increased the count and probability of TA occurring (figure 6*a*); however, no definitive pattern emerged which correlated with the length-scale itself. Rather, any introduction of heterogeneity at any length-scale increased the TA count relative to the homogeneous condition: mean TA count for the homogeneous model was 0.20 ± 0.06, compared to 0.44 ± 0.07 for *λ* = 1 µm (*p* < 0.05), 0.36 ± 0.07 for *λ* = 3 µm (*p* < 0.05), 0.58 ± 0.08 for *λ* = 5 µm (*p* < 0.01) and 0.5 ± 0.08 for *λ* = 10 µm (*p* < 0.01). When SCRE did occur, the mean magnitude of the spontaneous CaT (SCaT) did not differ significantly between different length-scales. However, the magnitude did vary significantly between individual maps, both within and between length-scales (figure 6*a*).

In contrast with condition 1, in condition 2 (ISO only, figure 6*b*) heterogeneous SERCA2a expression yielded a significant decrease in mean peak SCaT magnitude as length-scale increases. No significant differences were observed in the TA count for short length-scales (corresponding to control parameters, *λ* = 1, 3 µm), but at length-scales corresponding to HF cells (*λ* = 5, 10 µm), a significant reduction in the TA count was observed, from 0.55 ± 0.05 in.the homogeneous condition to 0.32 ± 0.1 for *λ* = 5 µm (*p* < 0.05) and 0.05 ± 0.04 for *λ* = 10 µm (*p* < 0.01).

There are two primary candidate mechanisms for heterogeneous SERCA2a expression influencing the nucleation and propagation of spontaneous Ca^2+^ waves: (i) the direct impact of local SERCA2a efflux, affecting the magnitude of Ca^2+^ which propagates to neighbouring CRUs; and (ii) the secondary effect of local SERCA2a activity determining local SR-Ca^2+^ load, which itself influences the probability of spontaneous or triggered Ca^2+^ sparks. These two mechanisms were isolated by performing simulations in which, following pacing to steady state, either homogeneous SR-Ca^2+^ load was imposed across the cell while maintaining the heterogeneous SERCA2a map, or the homogeneous SERCA2a map was imposed across the cell while maintaining the heterogeneous distribution of SR-Ca^2+^ load. These data (figure 6*c*) reveal that it is primarily the heterogeneous localized SR-Ca^2+^ load which determines the changes to SCRE activity, rather than the direct impact of SERCA2a on Ca^2+^ wave propagation. This explains the requirement of increased global SERCA2a for an increase in TA in heterogeneous maps, as it is these conditions which sufficiently load local regions of the SR-Ca^2+^ to both induce and maintain spontaneous Ca^2+^ waves; in the ISO-only condition, the loss of SERCA2a function and reduced whole-cell SR-Ca^2+^ load associated with increased *λ* (figure 2) is not sufficiently compensated by local regions of high SR-Ca^2+^, and SCRE are reduced.

There is a causal but complex correlation between local SERCA2a expression and the nucleation sites for spontaneous Ca^2+^ waves. As with normal pacing and alternans, regions of low-SERCA2a expression, and in particular those adjacent to regions of high SERCA2a expression, exhibit the largest SR-Ca^2+^ concentrations during diastole, and it is these regions which initially nucleate Ca^2+^ waves. However, following a whole-cell spontaneous release event, secondary events may be nucleated in similar locations (low SERCA2a adjacent to high SERCA2a) but may also originate from opposing locations (i.e. high SERCA2a), as a consequence of more rapid refilling in these regions during the Ca^2+^ wave (electronic supplementary material, figure S7).

## 4. Discussion

### (a) Summary of main findings

In this study, the correlation length-scale of SERCA2a expression in rat ventricular myocytes was quantified for the first time, to our knowledge, using a variogram fitting protocol (figures 1 and 2), demonstrating an increase in length-scale and inter-cellular variability in RV-HF. Simulations predict that increased SERCA2a heterogeneity results in reduced whole-cell CaT magnitude and more spatially disordered CaTs compared to the homogeneous models (figure 3). These cell-average changes were explained by a whole-cell drop in SERCA2a function (electronic supplementary material, figures S2 and S3). Furthermore, pro-arrhythmogenic behaviour was analysed across a large range of heterogeneous maps against a homogeneous control. Our simulations illustrated an increased propensity for spontaneous Ca^2+^ release events and incidences of spontaneous transmembrane depolarizations in the heterogeneous models (figure 6), which were demonstrated to be primarily owing to heterogeneous SR-Ca^2+^ loading. Several alternans behaviours were observed, with heterogeneous expression maps either promoting or inhibiting alternans depending on the environmental conditions (figure 5). Throughout, it is clear that inter-cellular variability of SERCA2a expression profile contributes to inter-cellular variability of Ca^2+^ dynamics, both during normal pacing and pro-arrhythmogenic conditions, and can partly explain the emergence of pro-arrhythmogenic cellular phenomena in RV-HF.

### (b) Implications for Ca^2+^-induced-Ca^2+^-release and contractile performance

HF is associated with a loss of contractile performance, underlain by reduced efficacy of CICR. The function of SERCA2a is strongly correlated with a decrease in SR-Ca^2+^ uptake in failing human hearts [35] and proposed as a causal factor for reduced CICR. Studies have provided mixed conclusions regarding whether SERCA2a expression is downregulated in the failing human heart, with some studies reporting no changes in HF [36–38], some observing a downregulation [35], whereas others have found a reduction in some cell types, but not others [39]. Other mechanisms have been proposed for a reduction in the SR-Ca^2+^, such as increased SR-Ca^2+^ leak through the RyRs [2] or inositol 1,4,5-triphosphate receptors (IP_3_Rs) [40]. This present study indicates that structural remodelling of SERCA2a (i.e. changes to its sub-cellular spatial profile) can, at least in-part, explain this loss of CICR, without any required changes to the global/whole-cell expression: length-scales of SERCA2a expression were significantly increased in RV-HF, and an increase in length-scale was strongly correlated with a reduction in diastolic SR-Ca^2+^ load and reduced magnitude of the CaT, as well as an increase in the spatial heterogeneity of the CaT itself. Moreover, heterogeneous SERCA2a expression resulted in an increase in inter-cellular variability, another feature commonly associated with HF [41,42]. Our analysis suggests that this reduction in diastolic SR-Ca^2+^ is a consequence of reduced activity of intracellular uptake in heterogeneous conditions owing to a combination of the inherent nonlinearities in the dependence of *J*_up_ on intracellular- and SR-Ca^2+^, and the impact of intra-SR diffusion.

### (c) Implications for Ca^2+^ transient alternans

Previous studies have shown the importance of CRU coupling and the inherently random dynamics of sub-cellular CaT alternans [34,43], which can be described as an order–disorder phase transition [44]. In another previous study [7], it was demonstrated that specific features of cellular geometry (e.g. proximity of cleft clusters; presence or absence of SR/T-system) reduced the randomness in which regions of the cell activate on subsequent large or small beats (i.e. the spatial phase variation was reduced).

The present study adds to this discussion by also demonstrating that heterogeneous magnitude of SERCA2a in different regions of the cell can also constrain the random spatial nature of CaT alternans, suggesting a shift of mechanism from the 3Rs described by Qu *et al*. [34] (which applies in homogeneous cells) to a more direct local Ca^2+^ dependence. This leads to largely the same spatial pattern of the CaT on subsequent small beats, contrary to what is observed in homogeneous cells. This difference in the fundamental underlying mechanisms of CaT alternans may have critical implications for the most effective and safe method to manage these phenomena.

Simulation results also highlight the sensitivity of the emergence of alternans to cellular conditions and reveal that introducing heterogeneity can critically shift the phase-space of the cell either into or out of an alternans producing region. It is unclear whether this is arrhythmogenic (shifting HF cells into pro-alternans phase-space), protective (a response to alternans by shifting HF cells out of the pro-alternans phase-space) or both (either through increased inter-cellular variability, or at different time-points of the progression of the disease). It will be important to establish whether remodelling of SERCA2a heterogeneity precedes, follows, or is concomitant with remodelling of whole-cell channel expression.

### (d) Implications for spontaneous arrhythmia triggers

HF is generally associated with increased cellular triggers, which may manifest as focal excitations in whole-heart-inducing arrhythmia [45]. A reduction in *I*_K1_ is observed in HF and promotes the emergence of TA from underlying SCRE [27,46]. However, this present study did not implement any changes to the ion-current expression and instead isolated the impact of changes to sub-cellular heterogeneity in SERCA2a. Whether such changes can underlie an increase in TA was not clear from the present study, and critically depended on the extent of SERCA2a upregulation used to promote SR-Ca^2+^ loading: above a threshold, increased length-scale (as observed in HF) was associated with an increase in SCRE and TA count; below this threshold, an increase in length-scale inhibited the emergence of TA. In either case, inter-cellular variability in the emergence of TA was substantially increased. HF conditions, such as fibrosis and reduced *I*_K1_, may significantly reduce the minimal substrate required for cellular TA to manifest in tissue and thus the increased presence of individual cells which are pro-TA could underlie increased arrhythmia triggers in HF. Further investigation is required at the systems level to determine whether an increase in SERCA2a heterogeneity in HF contributes to increased arrhythmia triggers.

The observed complex and biphasic impact of SERCA2a on SCRE is consistent with previous modelling and experimental studies [47–49], which have shown that increases in SERCA2a can both promote Ca^2+^ waves (through increased SR-Ca^2+^ load) but also inhibit them (through impairing inter-CRU Ca^2+^ propagation and increasing the SR threshold).

### (e) Limitations

There are a number of limitations associated with the present study, pertaining to the experimental data analysis and the simulation results. There is inherent spatial variation present within the imaging datasets owing to the quality of staining and differences in imaging conditions which may contribute towards estimation of correlation length-scale. This is mitigated by the down-sampling procedure which averages out this data over a resolution 10–50 times larger than the original image (figure 1*a*). To ensure no differences owing to imaging modality, only confocal microscopy images produced by the authors were used in this study.

The variogram fitting procedure works better with larger datasets; owing to the processing required by the image analysis step, some of this data is lost. This included condensing the data into two-dimensional, motivated by the variability in image quality and cell morphology, as well as the limited availability of data with a sufficient number of slices. To ensure reliable values were obtained from this analysis, each cell was analysed three times, each time requiring 50 successful variogram fits using a range of binning parameters suitable for each dataset (figure 1*b*). The final values of correlation length-scale are a statistical mean and standard error for each cell. Approximately 40% of the cells in this study were single images, with 60% having six or more images, and 30% having 20 or more. All cells analysed for this study were done so to the maximum possible extent; *z*-axis integration ensured both single images and stacks were comparable while ensuring three-dimensional features were captured. All images within a stack underwent the same processing step determined suitable for all images within that stack. This method may also measure anisotropy within sub-cellular expression; however, owing to the sizes of processed datasets, there was lower confidence in the estimations for anisotropy for the cells analysed in this study. For this reason, only isotropic analysis was considered in this study. It is likely that longitudinal-transverse anisotropy is a feature in sub-cellular heterogeneous expression at the micron scale, and this may contribute to the large error sizes in the cells with a higher correlation length-scale.

One major component which was not accounted for in the present study is the SERCA2a inhibitor phospholamban (PLB). The intracellular uptake flux, *J*_up_, is ultimately regulated by both SERCA2a and PLB expression, and therefore the assumption that local SERCA2a expression directly correlates with *J*_up_ magnitude is an over-simplification. It would be more correct to state that the heterogeneity maps implemented in the simulations represent *J*_up_ rather than SERCA2a. It would therefore be valuable in future studies to generate these maps based on combined analysis of SERCA2a and PLB, as it is unknown whether their heterogeneity will spatially correlate.

Four correlation length-scales (*λ* = 1, 3, 5 and 10 µm) were chosen for the computational study as they represented the range of heterogeneity observed in the image analysis study. Observing the full range of integer length-scales may have provided a smoother gradient of behaviour in length-scale; however, owing to the scope of this project, computational tractability and the range covered by this choice of length-scales, it was determined that they were sufficient to reveal the full range of emergent behaviour. Similarly, the total extent of heterogeneity was not varied within the present study, and expression was assumed to follow a normal distribution; it would be important to see if (and in what way) the impacts of length-scale are affected by both the total heterogeneity and the skew of the distribution.

The present study analysed myocytes only from healthy and RV-HF conditions, indicating that sub-cellular heterogeneity is a remodelled feature in HF. It will therefore be important to establish if this feature is present in other forms of HF (e.g. LV-HF or HF with preserved ejection fraction) and other pro-arrhythmogenic conditions, such as atrial fibrillation and ageing. It is noteworthy that the observed differences between control and HF are very similar in both RV and LV, despite this being an RV-HF model, indicating that this could possibly be a general and common feature of HF and perhaps other diseases.

The present study implemented models and data from multiple species, i.e. using experimental data from rat and a human-based computational model. This was motivated by the fundamentally mechanistic aims of the study in combination with the models of human ventricular electrophysiology being more robust and better developed than those of the rat. We note that in simulations, cell-specific heterogeneity maps were not used. Rather, maps at different length-scales were implemented, covering the range observed in the data, enabling the general mechanistic relationship between length-scale and dynamics to be elucidated. Future studies which aim to provide cell-specific insight, for example, in explaining specific functional data, would be better performed using data and models from the same species.

Whereas the present study focussed on heterogeneity at the macroscopic (micron) scale, super-resolution (nanometre) properties of heterogeneity and variability, such as clustering and co-localization distances with other channels, will also probably be highly important for governing local function [50]. Moreover, by isolating the impact of SERCA2a heterogeneity, the full systems perspective is somewhat missed. It will be important in future studies to combine SERCA2a heterogeneity with heterogeneity in other sub-cellular Ca^2+^ handling transporters (such as NCX and RyRs), as this will undoubtedly influence local flux balance and SR-Ca^2+^ loading, as well as in combination with the global remodelling of Ca^2+^ handling and ion-current channel expression. Furthermore, translating the impact on inter-cellular variability into tissue models would provide more substantial insight into the impact of SERCA2a heterogeneity and increased inter-cellular variability on the emergence of arrhythmia.

## 5. Conclusion

The present study has quantified the remodelling of SERCA2a sub-cellular heterogeneity in RV-HF. It demonstrates a general increase in the correlation length-scale, and its inter-cellular variability, with HF. These changes were predicted to contribute to reduce CICR under normal pacing conditions, as well as modulating, sometimes critically, the emergence of Ca^2+^-transient alternans and spontaneous Ca^2+^-release. We have therefore established that the spatial profile of SERCA2a in the sub-cellular volume, and potentially that of other Ca^2+^ handling transporters, is a property which may be remodelled in cardiovascular disease and can contribute to observed pathophysiology of function.

## Data Availability

Model code and heterogeneous SERCA2a maps are available at Michael Colman's Github repository: https://github.com/michaelcolman/MSCSF (maps are found in the folder MSCSF_Distribution/MSCSF/MSCSF_state_and_geometry_files if you already have the code). Data is also available in the electronic supplementary material [51].
